# Nucleosome Assembly and Disassembly *in vitro* Are Governed by Chemical Kinetic Principles

**DOI:** 10.3389/fcell.2021.762571

**Published:** 2021-10-07

**Authors:** Hongyu Zhao, Mingxin Guo, Fenghui Zhang, Xueqin Shao, Guoqing Liu, Yongqiang Xing, Xiujuan Zhao, Liaofu Luo, Lu Cai

**Affiliations:** ^1^School of Life Science and Technology, Inner Mongolia University of Science and Technology, Baotou, China; ^2^Inner Mongolia Key Laboratory of Functional Genome Bioinformatics, Inner Mongolia University of Science and Technology, Baotou, China

**Keywords:** nucleosome reconstitution *in vitro*, nucleosome disassembly, chemical kinetic model, nucleosome structure, nucleosome dynamics

## Abstract

As the elementary unit of eukaryotic chromatin, nucleosomes *in vivo* are highly dynamic in many biological processes, such as DNA replication, repair, recombination, or transcription, to allow the necessary factors to gain access to their substrate. The dynamic mechanism of nucleosome assembly and disassembly has not been well described thus far. We proposed a chemical kinetic model of nucleosome assembly and disassembly *in vitro*. In the model, the efficiency of nucleosome assembly was positively correlated with the total concentration of histone octamer, reaction rate constant and reaction time. All the corollaries of the model were well verified for the Widom 601 sequence and the six artificially synthesized DNA sequences, named CS1–CS6, by using the salt dialysis method *in vitro*. The reaction rate constant in the model may be used as a new parameter to evaluate the nucleosome reconstitution ability with DNAs. Nucleosome disassembly experiments for the Widom 601 sequence detected by Förster resonance energy transfer (FRET) and fluorescence thermal shift (FTS) assays demonstrated that nucleosome disassembly is the inverse process of assembly and can be described as three distinct stages: opening phase of the (H2A–H2B) dimer/(H3–H4)_2_ tetramer interface, release phase of the H2A–H2B dimers from (H3–H4)_2_ tetramer/DNA and removal phase of the (H3–H4)_2_ tetramer from DNA. Our kinetic model of nucleosome assembly and disassembly allows to confirm that nucleosome assembly and disassembly *in vitro* are governed by chemical kinetic principles.

## Introduction

The nucleosome is the elementary repeating unit of chromatin in eukaryotes. Approximately 147 base pairs (bp) of DNA in a left-handed superhelix wrap approximately 1.75 turns on an octamer containing two copies of four histone proteins to form a nucleosome core (Luger et al., 1997). In addition to serving as the building blocks of chromatin to pack DNA, nucleosome structure can also dynamically regulate many biological processes, such as transcription, DNA replication, repair, and recombination (Völker-Albert et al., 2016; Liu et al., 2019).

Nucleosomes are highly dynamic *in vivo*. Eviction of histones and reconstruction of nucleosomes occur frequently upon chromatin rearrangement (Kameda et al., 2019). Nucleosome positioning is malleable and movable along the DNA (Lai and Pugh, 2017; Liu et al., 2018). These dynamic nucleosomes can be regulated by posttranslational modifications (PTMs), replacing of their component histones, histone chaperones interacting with nucleosomes and remodeling devices. Gene expression involves nucleosomal rearrangement. In turn, changes in nucleosome positioning can also modulate gene expression by adjusting the DNA accessibility of regulatory proteins (Kameda et al., 2019).

Several works have investigated the dynamic process of nucleosomes. Ranjith et al. (2007) presented a kinetic model based on *Xenopus* egg extract solutions without added adenosine triphosphate (ATP) to describe the force-dependent on- and off-kinetics for nucleosomes and diffusion of nucleosomes along DNA. Förster resonance energy transfer (FRET) assays showed that the steps of nucleosome disassembly include the opening of the (H3–H4)_2_ tetramer/(H2A–H2B) dimer interface, H2A–H2B dimer release from the DNA and (H3–H4)_2_ tetramer removal (Gansen et al., 2009; Böhm et al., 2011). Remodeling kinetics models described the dynamics of chromatin remodeling driven by chromatin remodelers (Padinhateeri and Marko, 2011; Florescu et al., 2012). However, the intrinsic kinetics of the nucleosome assembly reaction without any chaperone remain elusive.

In view of the complexity of participation factors in nucleosome dynamics and the detection difficulty of nucleosome assembly and disassembly *in vivo*, nucleosome reconstitution *in vitro* by salt dialysis is the ideal model to elucidate the dynamic characteristics of nucleosome assembly and disassembly. Nucleosome assembly and disassembly by salt dialysis is not a strictly reversible chemical reaction. Based on the kinetic theory of chemical reactions, we proposed a chemical kinetic model to describe nucleosome assembly by salt dialysis *in vitro*. In the model, the efficiency of nucleosome assembly was positively correlated with the total concentration of histone octamer, reaction rate constant and reaction time. The reaction rate constant in the model may be used as a new parameter to evaluate the affinity of DNA to histones. The model was well tested for the Widom 601 sequence and the six artificially synthesized sequences, named CS1–CS6, by the salt dialysis method *in vitro*. Nucleosome disassembly experiments using the Widom 601 sequence detected by FRET and fluorescence thermal shift (FTS) assays demonstrated that nucleosome disassembly is the inverse process of assembly and can be described as three distinct stages: the opening phase of the (H2A–H2B) dimer/(H3–H4)_2_ tetramer interface, the release phase of the H2A–H2B dimers from (H3–H4)_2_ tetramer/DNA and the removal phase of the (H3–H4)_2_ tetramer from DNA. The present work elucidated that nucleosome assembly and disassembly *in vitro* are governed by chemical kinetic principles, and could provide deeper insight into the mechanism of nucleosome dynamics *in vivo*.

## Materials and Methods

### Preparation of DNAs and Recombinant Histone Octamer

To investigate the relation between N/S and underlying factors in assembly, 147-bp- length 601 DNA was labeled with Cy3 for canonical gel detection of nucleosomes (Lowary and Widom, 1998). The forward primer of polymerase chain reaction (PCR) was 5′-Cy3-CAGGATGTA TATATCTGACACGTGCCT-3′, and the reverse primer was 5′-CTGGAGAATCCCGGTGCCGAGGCC-3′. In addition, six artificially synthesized CS1–CS6 DNA sequences were used for experimental verification. Detailed sequence information is shown in our previous paper (Zhao et al., 2019). The forward primer of PCR was 5′-Cy3- ACGGCCAGTGAATTCGAGG-3′, and the reverse primer was 5′- GCCAAGCTTCTGAGATC GGAT-3′.

To reveal the nucleosome disassembly phases using gel electrophoresis and FRET analysis, 169-bp-long Widom 601 DNA fragments labeled by Cy3 and Cy5 of double fluorescence molecules with a Förster distance of ∼54 Å were prepared by PCR from a plasmid containing the 601 sequence. Forward primer: 5′-ACAGTACTGGCCGCCCTGGAGAATCCCGGTG CCGAGGCCGC***T***(Cy3)CAATTG-3′; reverse primer: 5′-TAC ATGCACAGGATGTATATATCTGACACGTGCCTGGAGAC***T*** (Cy5)AGGGAG-3′.

To understand the disassembly mechanism using FTS, 147-bp-long 601 fragments without any labeling marker were prepared by PCR from a plasmid containing the 601 sequence. The forward primer of PCR was 5′-CAGGA TGTAT ATAT CTGACA CGTGCCT-3′, and the reverse primer was 5′-CTGGA GAATC CCGG TGC CGAGGCC-3′.

All primers were synthesized in Sangon Biotech, China.

The expression and purification of histones were performed as described previously (Zhao et al., 2015, 2019). Briefly, four histones (H2A, H2B, H3, and H4) were expressed and purified from *Escherichia coli* BL21 cells containing pET-histone expression plasmids. To reconstitute the histone octamer, four histones with equimolar ratios were mixed in refolding buffer (2 M NaCl, 10 mM Tris–HCl, pH 7.5, 1 mM Na-EDTA, and 5 mM 2-mercaptoethanol). Histone octamers were purified through a Superdex S200 filtration column (GE Healthcare). Confirmation of the purity and stoichiometry of the histone octamers was performed using SDS-PAGE on 15% gels with Coomassie Brilliant Blue staining, and the concentration was determined using an extinction coefficient at 276 nm.

### Nucleosome Assembly Reaction *in vitro*

For *in vitro* structure investigation, mononucleosomes were assembled by using the salt-dialysis method as described previously (Zhao et al., 2015, 2019). Each DNA fragment was incubated in reconstitution reactions containing 10 mM Tris–HCl (pH 8.0), 1 mM EDTA (pH 8.0), 2 M NaCl, and histone octamers. The samples were placed in a microdialysis apparatus with 6–8 kDa dialysis tubing (Thermo Scientific, Slide-A-Lyzer MINI Dialysis Units, 7,000 MWCO). Then, they were placed in a beaker containing high-salt buffer (10 mM Tris–HCl, pH 8.0, 2 M NaCl, and 1 mM EDTA), which was continuously diluted by slowly pumping in TE buffer (10 mM Tris–HCl, pH 8.0, 1 mM EDTA) to a lower concentration of NaCl from 2 to 0.6 M over a period of 16 h. After this period, the samples were further dialyzed for an additional minimum of 3 h in TE buffer (10 mM Tris–HCl, pH 8.0, 1 mM EDTA) for gel analysis or in 10 mM HEPES buffer for FTS and FRET analysis. Dialysis was performed in a darkroom for the assembly reaction on fluorescence-labeled DNA templates. All of the steps were performed at 4°C.

In the reaction system, 3 μg DNA templates in total 60 μL reaction volume was used to assemble nucleosome. The molar concentration of 601 DNA sequence is 5.09 × 10^–7^ mol/L, and the molar concentration of CS DNA sequences is 4.62 × 10^–7^ mol/L in reaction system. As shown in Table 1, the concentrations of histone octamer and the ratios of histone octamer to DNA in reaction system have a change of gradient.

**TABLE 1:** The concentrations of histone octamer and the ratios of histone octamer to DNA in reaction system.

Mass concentrations of histone octamer (μg/mL)	Molar concentrations of histone octamer (mol/L)	Molecular ratio of histone octamer to 601 sequence	Molecular ratio of histone octamer to CS sequences
5	0.46 × 10^–7^	0.090	0.100
10	0.92 × 10^–7^	0.181	0.199
15	1.38 × 10^–7^	0.271	0.299
20	1.84 × 10^–7^	0.361	0.398
25	2.30 × 10^–7^	0.452	0.498
30	2.76 × 10^–7^	0.542	0.597
35	3.22 × 10^–7^	0.633	0.697
40	3.68 × 10^–7^	0.723	0.797
45	4.14 × 10^–7^	0.813	0.896
50	4.60 × 10^–7^	0.904	0.996
55	5.06 × 10^–7^	0.994	1.095
60	5.52 × 10^–7^	1.084	1.195

### Gel Analysis of Nucleosome Assembly Efficiency

For Cy3-labeled DNA templates, the reaction mixtures were resolved on 5% native polyacrylamide gels in 0.5 × TBE. The Cy3 fluorescence of nucleosome DNA and the free DNA band in the gel was measured and quantified at an emission wavelength of 605 nm and excitation wavelength of 520 nm (GE Healthcare, Amersham Imager 600RGB and Image Quant TL).

While Widom 601 DNA templates were labeled by Cy3 and Cy5 of double fluorescence molecule, the Cy5 fluorescence signal of nucleosome DNA and free DNA band in the gel was detected and quantified at an emission of 705 nm and excitation of 630 nm (GE Healthcare, Amersham Imager 600RGB and ImageQuant TL).

For nonlabeled DNA templates in FTS analysis, the reconstituted samples were loaded on 5% native polyacrylamide gels in 0.5 × TBE and stained with ethidium bromide.

### Förster Resonance Energy Transfer Analysis

The double-fluorescence-labeled Widom 601 DNA templates were reconstituted into mononucleosomes using the salt dialysis method as described above. FRET experiments were performed at 20°C on a fluorescence spectrometer (Bio-Tek, Cytation5).

For the temperature-dependent dissociation detection by FRET assay, reconstituted samples with different incubation time at 70°C were detected. The difference in fluorescence intensity between the donor and acceptor emissions was normalized. Then, the temperature-dependent dissociation curves of nucleosomes were generated between fluorescence intensity and incubation time.

For the salt-dependent dissociation study, samples with different concentrations of NaCl were excited at 485 nm, and the emission was recorded from 570 to 800 nm. The difference in the fluorescence intensity between the donor and acceptor emissions was plotted against the concentration of NaCl, which generated the salt-dependent dissociation curves of nucleosomes (Chen et al., 2013). The change rate of fluorescence was calculated by the difference of fluorescence against difference of ion concentration.

### Thermal Stability Assay

The stabilities of nucleosome dissociation were evaluated by a thermal stability shift assay as described previously (Sueoka et al., 2017; Arimura et al., 2018). A 147-bp-long 601 fragments without any labeling-marker were used. The thermal stability assay was performed in a solution containing, 0.25 M NaCl, 10 mM HEPES, 1 mM β-mercaptoethanol, and 5 × SYPRO Orange. The nucleosomes were equivalent to 375 ng DNA in each reaction. The total volume was adjusted to 30 μL.

The fluorescence signals of SYPRO Orange were recorded in the VIC channel of real-time PCR detection system (ABI 7500), and a temperature gradient was used from 25 to 95°C at each 1°C.

Raw fluorescence intensity data were normalized using the formula of $N\,F_{i}=\frac{F_{i}-F_{m\,i\,n}}{F_{m\,a\,x}-F_{m\,i\,n}}$, where *F*_*i*_, *F*_*min*_, and *F*_*max*_ indicate each fluorescence at a certain temperature, minimum and maximum of fluorescence intensity, respectively. The change rate of fluorescence was calculated by the formula of $C\,F_{i}=\frac{N\,F_{i+1}-N\,F_{i}}{T_{i+1}-T_{i}}$, where NF and *T* indicate normalized-fluorescence and temperature, respectively. The temperature range is 55–95°C.

## Results

### A Chemical Reaction Kinetics Model of Nucleosome Assembly

The nucleosome assembly reaction consists of three stages *in vitro*. First, the assembly and dissociation reaction of the histone octamer with its H3/H4 tetramer and two H2A/H2B dimers is not a strictly reversible process. Second, DNAs bind to H3/H4 tetramers to partially assemble an intermediate complex of nucleosomes. Third, two copies of the H2A/H2B dimer successively integrate into the intermediate complex to assemble a complete nucleosome. The above processes can be written in a set of reaction equations as follows.


(1) $$\{\begin{array}{c} P↔P_{4}+P_{2}+P_{2}^{\prime }\;\text{➀}\\ \begin{array}{c} D+P_{4}↔N_{a}\;\text{➁}\\ \begin{array}{c} N_{a}+P_{2}↔N_{b}\;\text{➂}\\ N_{b}+P_{2}^{\prime }↔N\;\text{➃} \end{array} \end{array} \end{array}$$

where *P*, *P*_4_, *P*_2,_ and $P_{2}^{\prime }$ represent the histone octamer, H3/H4 tetramer, and two H2A/H2B dimers, respectively. Na and Nb are two nucleosome intermediates. DNA molecules and the intact nucleosomes are denoted as *D* and *N*, respectively.

Using the mass action law of chemical reaction, we obtain a set of differential equations about the change of concentration of eight reaction components *P*, *P*_4_, *P*_2_, *P*_2_’, *D*, Na, Nb, and *N*. The equations are too complicated to obtain an analytical solution.

For the sake of simplicity, we propose a simplified model to describe the macrokinetics of the reaction process, namely, we study the overall reaction directly.


(2) D+P↔N

where the rate constants *k* and *k*’ of the forward and reverse reactions in Eq. 2 are assumed to be time-dependent, *k* = *k*(*t*) and *k*′ = *k*′(*t*),respectively. Using the same notation of molecule to represent its concentration, we obtain two differential equations on the concentration of nucleosomes, DNAs and histone octamers.


(3) $$\frac{d\,N}{d\,t}=k\,P\,D-k^{\prime }\,N$$

(4) $$\frac{d\,D}{d\,t}=\frac{d\,P}{d\,t}=-k\,P\,D+k^{\prime }\,N$$

where the total amount of DNA and histone octamer in the reaction system, named as *S* and *Q*, should be constant. Hence, we obtain Eq. 5


(5) $$\begin{array}{c} N+D=S\,\left(c\,o\,n\,s\,t\,a\,n\,t\right)\\ N+P=Q\,\left(c\,o\,n\,s\,t\,a\,n\,t\right) \end{array}$$

N/S is defined as the efficiency of nucleosome assembly. It is interesting to uncover the underlying factors affecting N/S and obtain analytic functions.

Combining Eqs. 3, 5, we obtain


(6) $$\frac{d\,N}{d\,t}=k\,N^{2}-\left(k^{\prime }+k\,S+k\,Q\right)\,N+k\,Q\,S$$

To integrate Eq. 6, one obtains


(7) $$\int_{0}^{N}\frac{d\,N}{N^{2}-\left(\frac{k^{\prime }}{k}+S+Q\right)\,N+Q\,S}=\int_{0}^{T}k\,dt$$

where *T* is the total dialysis time and the integral $\int_{0}^{T}k\,dt$ can be denoted as *θ* (*T*).

In the process of dialysis *in vitro*, *k*(t) and *k*’(t) are known since the concentration change of NaCl has been controlled. Thus, the integral in Eq. 7 can be calculated. In experiments, the total nucleosome reconstitution reaction can be split into several steps. The rate constant of the i-th step of the reaction is defined as *k*_*i*_, and the corresponding reaction time is denoted as τ_*i*_. One has $\theta =\sum_{i}k_{i}\,\tau_{i}=\bar{k}\,\sum \tau_{i}$ where $\bar{k}$ is the mean reaction constant and ∑τ_*i*_ = *T*_*ef*_ is the total efficient time of the reaction. The efficient time *T*_*ef*_ is an increasing function of the total dialysis time *T*. In our dialysis experiment, *T* changes in a relatively small range (approximately in the range from 0.01 to 0.02 h). Therefore, *T*_*ef*_ can be approximated as


(8) $$T_{e\,f}=\alpha +\varepsilon \,T$$

and we have


(9) $$\theta \,\left(T\right)=\int_{0}^{T}k\,dt=\bar{k}\,T_{e\,f}=\bar{k}\,\left(\alpha +\varepsilon \,T\right)$$

Considering that the gradient descent of NaCl concentration mainly promotes the nucleosome assembly, as a first order approximation, we assume *k*’ to be ignored in Eq. 7. We obtain:


$$\int_{0}^{N}\frac{d\,N}{N^{2}-\left(S+Q\right)\,N+Q\,S}=\frac{1}{Q-S}\,\left(l\,n\,\frac{N-Q}{N-S}-l\,n\,\frac{Q}{S}\right)=\theta \,\left(T\right)$$

It leads to


(10) $$\frac{N}{S}=\frac{\frac{Q}{S}\,\left\{1-e\,x\,p\,\left[\left(Q-S\right)\,\theta \,\left(T\right)\right]\right\}}{1-\frac{Q}{S}\,e\,x\,p\,\left\{\left(Q-S\right)\,\theta \,\left(T\right)\right\}}$$

Under (*Q* − *S*) *θ* (*T*) = 1, the exponential function in Eq. 10 is expanded to 2nd order of (*Q* − *S*)*θ*(*T*), and it follows


(10.1) $$\frac{N}{S}≅\frac{Q\,\vartheta \,\left(T\right)}{1+Q\,\vartheta \,\left(T\right)}=\frac{Q\,\bar{k}\,T_{e\,f}}{1+Q\,\bar{k}\,T_{e\,f}}=\frac{Q\,\bar{k}\,\left(\alpha +\varepsilon \,T\right)}{1+Q\,\bar{k}\,\left(\alpha +\varepsilon \,T\right)}$$

which shows N/S approaches to 1 as Q>>S and approaches to 0 as Q<<S. A simplified form of Eq. 10.1 for not-too-large Q is the linear relation between N/S and Q


(11) $$\frac{N}{S}=Q\,\bar{k}\,\left(\alpha +\varepsilon \,T\right)$$

which is useful in analyzing experimental data.

Otherwise, if *k*’ cannot be ignored in Eq. 7, then we obtain


$$\frac{1}{Q-S+\gamma \,\frac{Q+S}{Q-S}}\,l\,n\,\left(\frac{\left\{N-Q\,\left(1+\frac{\gamma }{Q-S}\right)\right\}\,\left\{S\,\left(1-\frac{\gamma }{Q-S}\right)\right\}}{\left\{Q\,\left(1+\frac{\gamma }{Q-S}\right)\right\}\,\left\{N-S\,\left(1-\frac{\gamma }{Q-S}\right)\right\}}\right)$$

(12) $$=\bar{k}\,\left(\alpha +\varepsilon \,T\right)$$

where γ is the integral median of (*k*′/*k*). As $\frac{\gamma }{\left|Q-S\right|}\ll 1$, Eq. 12 returns to Eq. 10.

Equation 12 gives a rigor expression of N/S depending on the total concentration of DNA and histone octamer, reaction rate constant and reaction time. Eq. 10 is a simplified representation under the condition of ignoring the disassembly reaction, and Eq. 11 provides a simplified linear relation for analyzing experimental data.

### The Efficiency of Nucleosome Assembly Is Proportional to Histone Concentration

**FIGURE 1:**
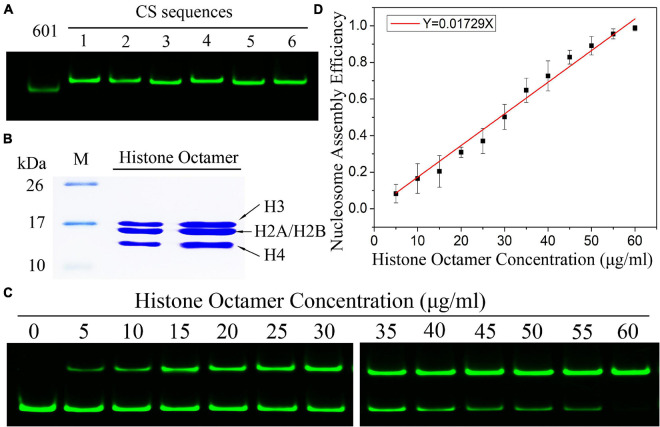
The efficiency of nucleosome assembly on the Widom 601 sequence *in vitro* by salt dialysis is dependent on histone concentration. **(A)** Preparation of Widom 601 DNAs and CS DNAs. The Cy3-labeled DNA sequences were detected by native-PAGE. **(B)** Preparation of histone octamer. The reconstituted canonical histone octamer was analyzed by SDS-PAGE. **(C)** Detection results of nucleosome assembly *in vitro*. The reconstituted nucleosomes with different histone octamers were analyzed by native PAGE. In each lane of the gel, the top band is nucleosome DNAs, and the bottom band is free DNAs. **(D)** The relation curves of nucleosome assembly efficiency vs. histone octamer concentration for the Widom 601 sequence. Nucleosome assembly efficiency was calculated by the ratio of nucleosome DNAs to total DNAs from panel **(C)** for each reconstituted sample. For each sample, five independent repeats were performed.

By using the nucleosome reconstitution method *in vitro* and the canonical gel detection of nucleosomes we shall test the relation between assembly efficiency N/S and histone concentration *Q* deduced from the chemical kinetic model. First, the Widom 601 DNA sequence (Lowary and Widom, 1998) was labeled by the fluorescence molecular probe Cy3 (Figure 1A). A recombinant histone octamer, that lacked all PTMs, was expressed and purified from bacteria (Figure 1B). Then, we assembled the mononucleosomes on 601 DNA templates with a concentration gradient of histone octamers. As shown in Figure 1C, after separation by gel electrophoresis, the nucleosome-assembled DNAs appeared as retarded bands compared to free DNAs. The ratio of nucleosome DNA in the assembled sample showed an increasing trend with the histone octamer concentration. We then quantified the ratio of nucleosome DNA to total DNA (N/S in Eq. 10) as an assembly efficiency to evaluate the nucleosome formation ability of each assembled sample. The nucleosome assembly efficiency was positively correlated with the histone octamer concentration (Figure 1D). This result of nucleosome assembly on the 601 DNA template *in vitro* indicated that N/S has a significant linear correlation with *Q* for not-too-large *Q*, which is consistent with Eq. 11.

Then, we examined the nucleosome assembly efficiency for six CS DNA sequences with a histone octamer concentration gradient (Supplementary Figure 1). CS DNA templates were designed with different sequence features in our previous work (Zhao et al., 2019). CS1 sequences consist of uninterrupted 11 copies of RRRRRYYYYY (named the R5Y5 motif, here *R* = purine, *Y* = pyrimidine), but do not contain a 10.5-bp periodicity of TA dinucleotides. CS2 and CS3 fit with 11 uninterrupted units of the R5Y5 motif and visible 10.5-bp periodicity of TA dinucleotides. Sequences CS4, CS5, and CS6 contain 10.5 bp periodic TA dinucleotides but do not contain the R5Y5 motif. We found that nucleosome assembly efficiency (N/S) is proportional to histone octamer concentration (Q) for CS1–CS6 at the same reaction condition and time, which is the same as that for the Widom 601 sequence.

### Parameter $\bar{k}$ Can Be Used to Evaluate the Affinity of DNA Fragments to Histone Octamers

In the model, parameter $\bar{k}$ is the mean reaction rate constant in the process of nucleosome assembly. The slope coefficient of linear fitting in Eq. 11 is $\varepsilon \,\bar{k}$, where ε should be a constant under the same experimental condition for the reconstituted reaction on six CS sequences. We may directly use the slope coefficient to evaluate the nucleosome formation ability with DNA sequences. To examine this hypothesis, we used six CS sequences to assemble mononucleosomes *in vitro* by salt-dialysis (Supplementary Figure 1). Our previous work demonstrated that CS2 and CS3 sequences containing both the R5Y5 motif and TA repeats with 10.5-bp periodicity have a stronger ability to assemble nucleosomes, and the CS1 sequence with only the R5Y5 motif has a lower affinity to histones *in vitro* among the six DNA sequences (Zhao et al., 2019).

**FIGURE 2:**
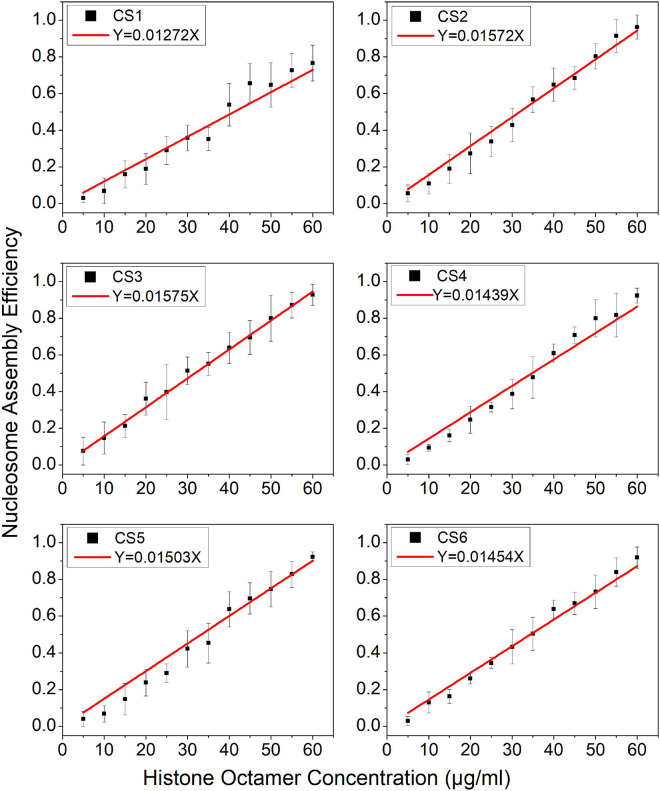
The regression curve of nucleosome assembly efficiency vs. histone octamer concentration for six CS sequences. The nucleosomes were reconstituted on CS1–CS6 DNAs with different histone octamer concentrations. Reconstituted nucleosomes were analyzed by native-PAGE and quantized to calculate the nucleosome assembly efficiency. For each sample, five independent repeats were performed.

The slope coefficients of linear fitting on CS2 and CS3 were significantly higher than those on other CS sequences (Figure 2, paired-sample *t*-test, *p* < 0.01), which suggested that CS2 and CS3 have a higher affinity for histone octamers. The slope coefficient on CS1 was only 0.01272, which was the lowest among the six CS sequences (Figure 2, paired-sample *t*-test, *p* < 0.01). These results were highly consistent with our previous report (Zhao et al., 2019) and suggested that the parameter $\bar{k}$ can be used to evaluate the affinity between histones and DNA sequences.

### The Efficiency of Nucleosome Assembly Is Proportional to Dialysis Time

**FIGURE 3:**
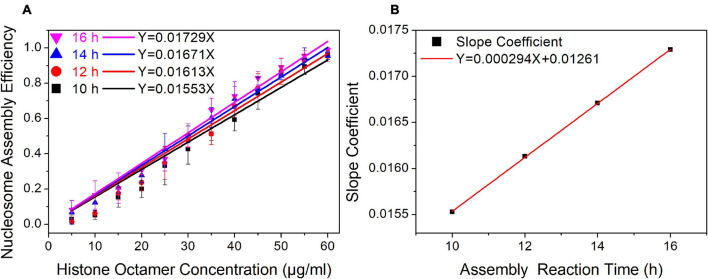
The regression curve of nucleosome assembly efficiency vs. reaction time for 601 DNA sequence under different assembly reaction times. **(A)** Regression curves of nucleosome assembly efficiency vs. histone octamer concentration under 10, 12, 14, and 16 h of dialysis time. For each sample, five independent repeats were performed. **(B)** Linear regression curve between the slope coefficient in panel **(A)** and assembly reaction time.

In our nucleosome assembly kinetics model, assembly reaction time was one of the parameters affecting the nucleosome assembly efficiency *in vitro*. We then assembled nucleosomes on a Widom 601 sequence with a concentration gradient of histone octamers under dialysis times of 10, 12, 14, and 16 h (Supplementary Figure 2). As shown in Figure 3A, the nucleosome assembly efficiency displayed a significant linear correlation with histone octamer concentration at four dialysis times (*p* < 0.01). Interestingly, the fitting curve between slope coefficient in Figure 3A and assembly reaction time showed a high linear dependence (Figure 3B, *p* < 0.01). This result confirmed the linear relationship between N/S and *T* in Eq. 11.

### Nucleosome Disassembly Can Be Described as Three Distinct Stages *in vitro*

In our initial reaction Eq. 1, nucleosome assembly contains two key stages: the binding of the (H3/H4)_2_ tetramer to DNA and the (H2A/H2B) dimer to (H3/H4)_2_/DNA. In salt dialysis, gradient descent of salty ions can promote the binding of negatively charged DNAs to histones. It is easier to understand the kinetic mechanism from nucleosome disassembly than from assembly.

**FIGURE 4:**
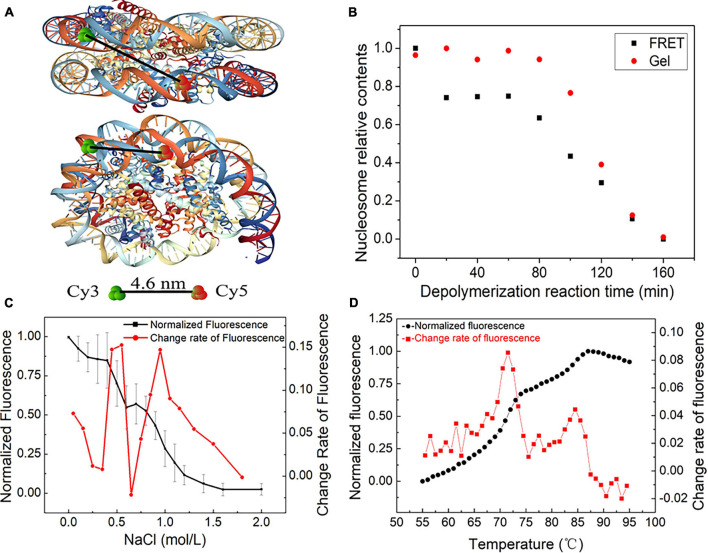
The dynamic stages of nucleosome disassembly *in vitro* using the Widom 601 sequence. **(A)** Top and side views of the nucleosome with the position of donor Cy3 and acceptor Cy5 (structural outline of nucleosome: PDB ID 3 L Z1). **(B)** Nucleosome disassembly after incubation at 70°C. Red dots denote the amount of nucleosome DNA in total DNA detected by native PAGE, and black dots denote the relative signals of Förster resonance energy transfer (FRET) generated in nucleosomes. **(C)** FRET analysis of NaCl-dependent dissociation of nucleosomes. The black dots denote the normalized FRET signals obtained by monitoring the fluorescence difference between the donor and acceptor emissions upon donor excitation at 480 nm. The red dots are the change rate of FRET signals to NaCl concentration. **(D)** Thermal shift assays with the nucleosome using SYPRO Orange. The relative fluorescence intensity at each temperature is plotted as a black dot. The red dots show the differential values of the thermal stability curves presented in the black dots.

We employed a FRET assay to monitor the dynamic change in nucleosome structure in the disassembly process. In this assay, we labeled Widom 601 DNA sequences with a donor Cy3 and an acceptor Cy5 over a 96-bp separation. Because of the over 30 nm length between the two dyes, FRET signals on free DNA templates cannot be detected (Supplementary Figure 3). While the nucleosome is reconstituted on the Widom 601 DNA template, the spatial distance of the two fluorescent molecules is reduced to approximately 4.6 nm (Figure 4A), which enables the well-organized nucleosome to be excited to produce efficient FRET signals (Supplementary Figure 3).

Nucleosome disassembly under high temperature was detected by FRET assay. The reconstituted nucleosomes were incubated at the temperature of 70°C. We used both native polyacrylamide gel electrophoresis (PAGE) and FRET to detect nucleosome disassembly. In the first 80 min of the disassembly reaction, the amount of nucleosome DNA in the total DNA showed no obvious change by PAGE (Figure 4B). In other words, we cannot detect the physical separation between DNA and histones in this stage. However, as the disassembly reaction started, the FRET signals quickly decreased and then remained unchanged for ∼60 min. These results implied that the DNA wrapping on the histone octamer has become loose, but the nucleosome does not depolymerize at this primary stage. After ∼80 min of incubation at 70°C, the amount of nucleosome DNA in PAGE detection decreased with increasing disassembly reaction time. FRET signals also display a similar downtrend at this quick disassembly stage. Combining the results of PAGE and FRET assays, one can conclude that relaxation of the nucleosome spatial structure begins before the physical separation of DNA from histones is completed.

Quantitative FRET signals were used to monitor the NaCl-dependent disassembly process of nucleosomes (Supplementary Figure 4). The normalized FRET signal has two rapid descent stages with increasing NaCl concentration (Figure 4C). Then, we calculated the change rate of fluorescence to NaCl concentration. Two obvious peaks of the change rate of fluorescence were observed at ∼0.6 and 1.0 mol/L NaCl (Figure 4C). This result suggested that H2A/H2B dimer disassembly (reverse reaction in Eq. 1-➂➃) and H3/H4 tetramer depolymerization (reverse reaction in Eq. 1-➁) may contribute to the first peak and second peak, respectively.

Then, we employed an FTS assay to detect thermal stability-dependent nucleosome disassembly. The nucleosomes were reconstituted on 147-bp 601 DNA templates without any fluorescence labeling. We then performed FTS experiments with SYPRO Orange. This method monitors the fluorescence signal from SYPRO Orange, which binds hydrophobically to the proteins by thermal denaturation. In this assay, the histones that thermally dissociate from the nucleosome are detected by fluorescent signals of SYPRO Orange (Arimura et al., 2018). As shown in Figure 4D, the fluorescence signal intensity began to increase significantly after 55°C, which suggested that the nucleosomes started to decompose. The first rapid increase in the fluorescence from 68 to 75°C indicates the removal of the H2A/H2B dimer, and the later peak from 83 to 87°C indicates the dissociation of the H3/H4 tetramer from DNA. These results also support staged characteristics of nucleosome disassembly in our initial reaction Eq. 1.

In summary, nucleosome disassembly can be described as three distinct stages: the opening phase of the (H3–H4)_2_ tetramer/(H2A–H2B) dimer interface, the release phase of H2A–H2B dimer from (H3–H4)_2_ tetramer/DNA and the removal phase of (H3–H4)_2_ tetramer from DNA.

## Discussion

In this work, we proposed a chemical kinetic model of nucleosome assembly. Nucleosome reconstitution assays by salt dialysis *in vitro* demonstrated that the efficiency of nucleosome assembly was positively correlated with the concentration of histone octamer, reaction rate constant, and reaction time in this model. All the conclusions of the kinetic model were well confirmed for selected sequences by using the salt dialysis method *in vitro*. Our theoretical model and experimental test reveal that nucleosome assembly and disassembly *in vitro* are governed by chemical kinetic principles.

In the derivation process of Eq. 11, we bring in hypothesis (*Q* − *S*)*θ*(*T*) ≪ 1 to expand the exponential function by the Taylor mean value theorem. For the extreme case of *Q* ≫ *S*, the condition (*Q* − *S*)*θ*(*T*) ≪ 1 is not met. While we take the limit of Eq. 10, $\frac{N}{S}=\frac{\frac{Q}{S}\,\left(e\,x\,p\,\left(-\left(Q-S\right)\,\theta \right)-1\right)}{e\,x\,p\,\left(-\left(Q-S\right)\,\theta \right)-\frac{Q}{S}}\approx 1$ can be obtained. In our reconstituted nucleosome assays, the supersaturated concentration of histone octamer in the reaction system can lead all DNAs to assemble nucleosomes, in other words, $\frac{N}{S}$ should be 1 in this case. On the other hand, for the extreme case of *Q* ≪ *S*, one can obtain $\frac{N}{S}=\frac{\frac{Q}{S}\,\left\{1-e\,x\,p\,\left[\left(Q-S\right)\,\theta \,\left(T\right)\right]\right\}}{1-\frac{Q}{S}\,e\,x\,p\,\left\{\left(Q-S\right)\,\theta \,\left(T\right)\right\}}\approx \frac{Q}{S}\approx 0$ in Eq. 10. While the concentration of histone octamers was far less than the concentration of DNAs, the DNAs could hardly reconstitute into nucleosomes. This experimental observation is consistent with the theoretical calculation result. Therefore, the condition (*Q* − *S*) *θ*(*T*) ≪ 1 is reasonable for the nucleosome assembly *in vitro*.

In a previous study, the affinity of DNAs to histones was usually quantified by the ratio of nucleosome DNA to free DNA in reconstituted nucleosome samples and/or the relative Gibbs free energy of reconstituted reaction under only one specific concentration of histone octamer (Thåström et al., 2004a; Volle and Delaney, 2012). However, the nucleosome assembly efficiency is associated with histone octamer. In the present kinetic model, reaction rate constant $\bar{k}$ is an important parameter. Comparing the difference of reaction rate constant $\bar{k}$among different DNAs is a systematic evaluation method for the affinity of DNAs to histone, which can provide a more comprehensive understanding from the reconstituted reaction under gradient concentration of histone octamer than previous method only from one concentration of histone octamer.

Our model describes the chemical kinetics of nucleosomes based on nucleosome assembly and disassembly assays *in vitro*. Nucleosome reconstitution, dissociation and remodeling *in vivo* are more complicated than that *in vitro*. Nucleosome assembly chaperonin and chromatin remodeler are intimately involved in the dynamics of nucleosomes *in vivo*. Our kinetics model may not be directly used to describe the apparent kinetics of nucleosome dynamics *in vivo*. However, the intrinsic kinetics, which only involve the interaction of DNAs and histones, may elucidate the basic rule in the kinetic principle of nucleosome assembly and could provide the ideal model to develop further an apparent kinetics model of nucleosomes.

Our experiments showed that nucleosome disassembly can be described as three distinct stages: opening phase of the (H2A/H2B) dimer/(H3/H4)_2_ tetramer interface, release phase of the H2A/H2B dimers from (H3/H4)_2_ tetramer/DNA and removal phase of the (H3/H4)_2_ tetramer from DNA. This result may be helpful for the understanding the effects of different physiological variables on dimers stability. H2A/H2B dimers dissociation can be crucial in the efficiency of transcription elongation, and the process *in vivo* is often regulated by transcription factor, such as FACT (facilitates chromatin transcription) complex. Hsieh et al. (2013) revealed that FACT can induce global accessibility of nucleosomal DNA without histone H2A/H2B displacement and thus can facilitate action of processive enzymes on DNA, such as transcription through chromatin. Chen et al. (2018) demonstrated that FACT displays dual functions in destabilizing the nucleosome and maintaining the original histones and nucleosome integrity at the single-nucleosome level. At early 1990s, researchers attempted to understand the mechanical behavior in the interaction between DNA and histones. DNA topological parameters, such as DNA linking variants, torsional stress, were used to elucidate mechanism of nucleosome structure (Negri et al., 1994; Negri and Di Mauro, 1997). PTMs in histone proteins play essential roles in nucleosome dynamics. The results from three-color single-molecule FRET showed that H2A/H2B dimer displacement process has a slight difference between in the salt-induced case and in the Nap1-mediated case. For the Nap1-mediated dimer dissociation, the acetylation at histone H4K16 or H3K56 facilitates the process both kinetically and thermodynamically (Lee and Lee, 2017). Sueoka et al. (2017) uncovered that phosphorylation at H2A Tyr57 changes the stability of the H2A-H2B dimer but does not interfere with histone-DNA interactions, an facilitate the dissociation of H2A/H2B dimer from the nucleosome structure. The acetylation and ubiquitination of histones H2A and H2B.1 weaken their interaction with the (H3-H4)2 tetramer and/or nucleosomal DNA, while histones H2A.Z and H2B.2 strengthen these interactions (Li et al., 1993). So far, how these complex factors regulate the H2A/H2B dimers dissociation *in vivo* is not fully understood.

Taking into account the irreversible of nucleosome assembly/disassembly process and the cooperative behavior of the increases in [NaCl] or temperature in salt-dialysis method, Thåström et al. (2004b) emphasized that equilibrium affinities cannot be obtained from these measurements. The chemical kinetics discussed in present work is regarded to the nucleosome assembly driven by NaCl dilution. As we know, different from equilibrium thermodynamics, the chemical kinetics generally discusses the time-dependent process and does not require the reversibility of the process. Experiments on increase in [NaCl] influencing assembly/disassembly are not the reversal process of that we discussed. Therefore, there is no conflict between Thåström’s work and our model.

The ATP-dependent assembly of periodic nucleosome arrays and the ATP-independent random deposition of histones onto DNA (such as salt-dialysis method) are two kinds of popular strategies in the reconstitution of chromatin *in vitro* (Lusser and Kadonaga, 2004). Some simplification is inevitable in nucleosome assembly system *in vitro*. The central question is whether this simplification can reveal the laws of nucleosome assembly. The ATP-dependent assembly reaction can produce periodic nucleosome arrays, similar to those seen in bulk native chromatin. This assembly method requires ATP-utilizing chromatin assembly factors, such as ACF (ATP-utilizing chromatin assembly and remodeling factor) or RSF (remodeling and spacing factor), etc., (Lusser and Kadonaga, 2004). Even so, this assembly reaction *in vitro* is still not a complete simulation of the complex nucleosome assembly *in vivo*. The salt-dialysis method, in which the reaction temperature and concentration gradient of saline ions is constant, is one of ATP-independent strategy to assemble the nucleosomes *in vitro*. The only biological molecules in this reaction system are DNA and histones. The nucleosome assembly efficiency is not affected by the other factors, such as chromatin remodelers, histone chaperones. Thus, the salt-dialysis method of nucleosome assembly can be regarded as a simple model of nucleosome assembly *in vivo*. In this work, more attention was paid on the affinity between DNA and histone octamer in nucleosome assembly reaction. So, employing the salt-dialysis method should be sounder and more feasible. We used the salt-dialysis experimental system *in vitro* to well uncover the relation between nucleosome assembly efficiency and DNA sequences, concentration of histone octamer, and reaction time. These results suggest that nucleosome assembly/disassembly *in vitro* is governed by chemical kinetic principles. This conclusion has merit for further understanding the nucleosome dynamics *in vivo*. In recent years, several studies revealed that nucleosome organizations *in vivo* are dominantly encoded in the genomic sequence and nucleosomes’ intrinsic DNA sequence preferences vary greatly between differing DNA sequences (Field et al., 2008; Kaplan et al., 2009). These results imply that the roughly nucleosome position *in vivo* can be determined by the affinity between DNA and histone octamer, which can be simulated by the salt-dialysis method of nucleosome assembly *in vitro*, and the accurate nucleosome position is modulated by other factors, such as histone chaperone, histone modification. Taking into account other factors, our further research will focus on the simulation of the nucleosome assembly *in vivo*.

In future, we can integrate DNAs, histone octamer, histone chaperones, and chromatin remodelers into a complex model for further understanding the mechanism of nucleosome assembly. In the model, we can investigate the effect of mechanical characteristics of DNAs, histone variables and physiological variables on nucleosome assembly. Meanwhile, a complex system of nucleosome reconstitution *in vitro* can be constructed by combining salt dialysis, histone chaperones and ATP-dependent assembly factors. This nucleosome reconstitution system can be used to examine more complicated factors in theoretical model. The new model may get closer to nucleosome dynamics *in vivo*. Further, we can also introduce RNA polymerase II into nucleosome assembly model and nucleosome assembly reaction system. By analyzing the competitive binding to DNAs of RNA polymerase II and histone, we can attempt to understand the coupling mechanism of transcription elongation and nucleosome dynamics.

Taken together, we propose a chemical kinetics model to describe the dynamic nucleosome assembly, and the results reveal that nucleosome assembly and disassembly *in vitro* are governed by chemical kinetic principles. We provide a novel evaluation method in which parameter $\bar{k}$ can be used to evaluate the affinity of DNAs to histones. In addition, we further confirmed that there exist three distinct stages in nucleosome dynamics, which is consistent with the conclusions of previous work (Gansen et al., 2009; Böhm et al., 2011). These results will contribute to further understanding the dynamics of nucleosomes *in vivo*.

## Data Availability Statement

The original contributions presented in the study are included in the article/Supplementary Material, further inquiries can be directed to the corresponding author/s.

## Author Contributions

LC, LL, and HZ designed the research, contributed to the theoretical model, and wrote the manuscript. HZ, MG, FZ, and XS performed the experiment. GL, YX, and XZ analyzed the data. All authors contributed to the article and approved the submitted version.

## Conflict of Interest

The authors declare that the research was conducted in the absence of any commercial or financial relationships that could be construed as a potential conflict of interest.

## Publisher’s Note

All claims expressed in this article are solely those of the authors and do not necessarily represent those of their affiliated organizations, or those of the publisher, the editors and the reviewers. Any product that may be evaluated in this article, or claim that may be made by its manufacturer, is not guaranteed or endorsed by the publisher.
