# Supplementary material for: Nucleosome Assembly and Disassembly in vitro Are Governed by Chemical Kinetic Principles
Source: Front Cell Dev Biol. 2021 Oct 7;9:762571. doi: 10.3389/fcell.2021.762571 (PMC8529108; doi:10.3389/fcell.2021.762571)
Supplement: Supplementary file 1 [file Data_Sheet_1.docx]

Supplementary Material

## Supplementary Figures


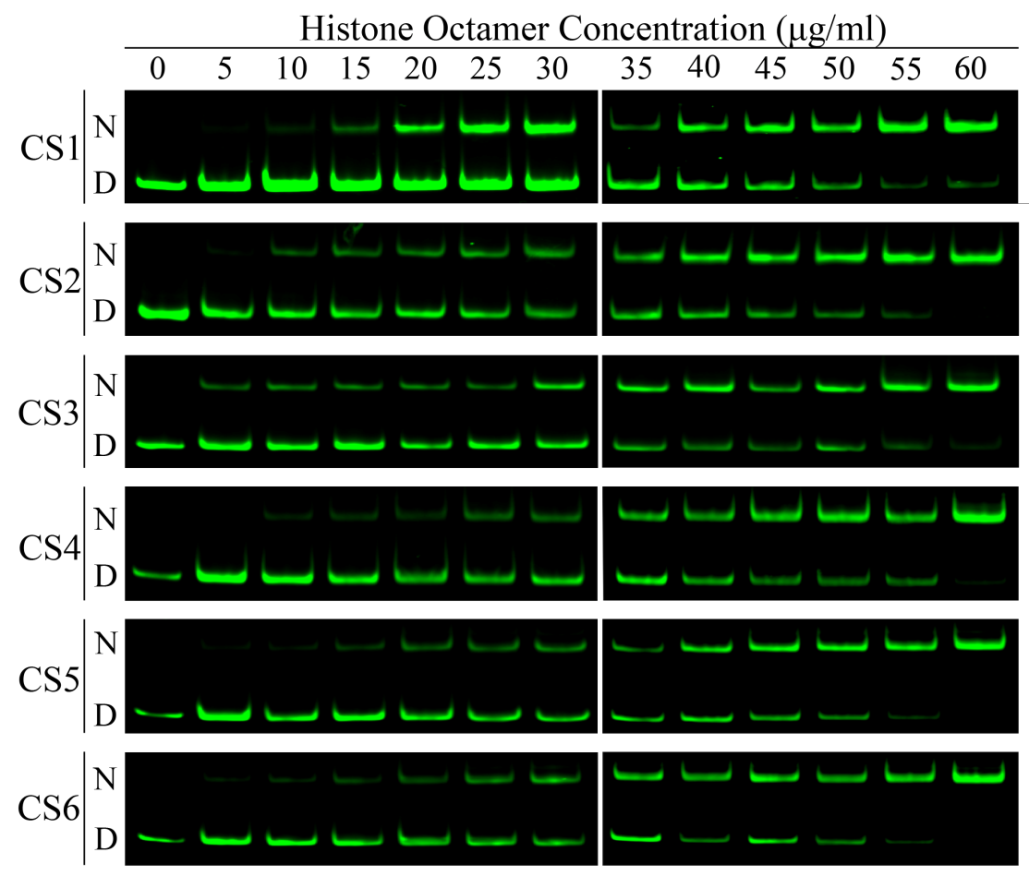


Supplementary Figure 1. Native PAGE results of reconstituted nucleosomes on Cy3-labeled CS1-CS6 DNA templates with different histone octamer concentrations. N denotes nucleosome DNA, and D denotes free DNA.


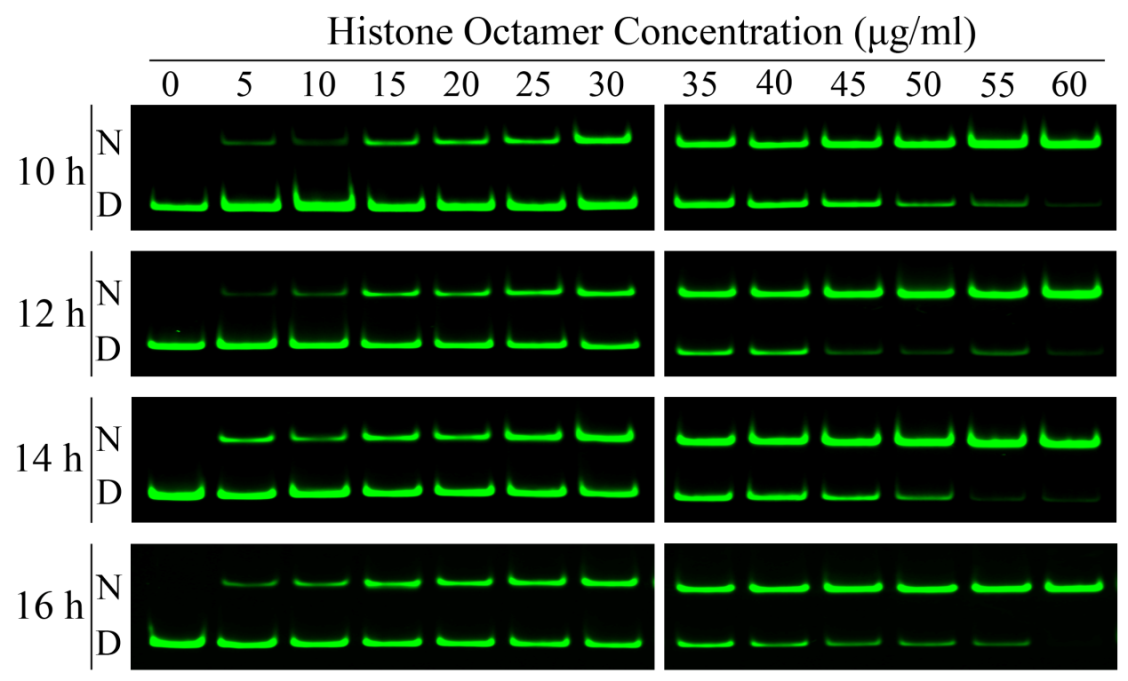


Supplementary Figure 2. Native PAGE results of reconstituted nucleosomes on Cy3-labeled Widom 601 DNA templates under dialysis times of 10 hours, 12 hours, 14 hours and 16 hours. N denotes nucleosome DNA, and D denotes free DNA.





Supplementary Figure 3. Fluorescence emission spectra of the primer, DNA and reconstituted nucleosomes upon donor excitation at 485 nm. The black curve denotes the emission spectra of Cy3-labeled primers, the red curve denotes the emission spectra of free DNA labeled by Cy3 and Cy5 fluorescent molecules, and the green curve denotes the emission spectra of nucleosomes reconstituted on labeled DNA by Cy3 and Cy5 fluorescent molecules.





Supplementary Figure 4. FRET analysis of NaCl-dependent dissociation of nucleosomes.
